# Benzo(a)pyrene in Cigarette Smoke Enhances HIV-1 Replication through NF-κB Activation via CYP-Mediated Oxidative Stress Pathway

**DOI:** 10.1038/s41598-018-28500-z

**Published:** 2018-07-10

**Authors:** Sabina Ranjit, Namita Sinha, Sunitha Kodidela, Santosh Kumar

**Affiliations:** Department of Pharmaceutical Sciences, University of Tennessee Health Science Center, Memphis, TN 38163 USA

## Abstract

Smoking aggravates HIV-1 pathogenesis and leads to decreased responses to antiretroviral therapy. In this study, we aim to find a molecular mechanism that would explain smoking-induced HIV-1 replication. Benzo(a)pyrene (BaP), a major carcinogen in cigarette, requires metabolic activation through cytochrome P450s (CYPs) to exert its toxic effects. We hypothesized that CYP-mediated BaP metabolism generates reactive oxygen species (ROS), and the resultant oxidative stress aggravates HIV-1 replication. As expected, we observed ~3 to 4-fold increase in HIV-1 replication in U1 cells and human primary macrophages after chronic BaP exposure. We also observed ~30-fold increase in the expression of CYP1A1 at mRNA level, ~2.5-fold increase in its enzymatic activity as well as elevated ROS and cytotoxicity in U1 cells. The knock-down of the CYP1A1 gene using siRNA and treatment with selective CYP inhibitors and antioxidants significantly reduced HIV-1 replication. Further, we observed a nuclear translocation of NF-κB subunits (p50 and p65) after chronic BaP exposure, which was reduced by treatment with siRNA and antioxidants/CYP inhibitors. Suppression of NF-κB pathway using specific NF-κB inhibitors also significantly reduced HIV-1 replication. Altogether, our results suggest that BaP enhances HIV-1 replication in macrophages by a CYP-mediated oxidative stress pathway followed by the NF-κB pathway.

## Introduction

The association of cigarette smoking and HIV-1 pathogenesis has been demonstrated by multiple studies in the past two decades^1–6^. Smoking increases HIV-1 infectivity and viral load, and it lowers the CD4 counts in HIV-1 patients, with a subsequent increase in immunosuppression^3,7^. Smoking also decreases the response to antiretroviral therapy (ART) by approximately 40% in HIV-1 patients^8^, which further accentuates the hazards of smoking on HIV-1 pathogenesis. However, little is known about the mechanisms underlying smoking-induced HIV-1 replication. A recent study has shown that cigarette smoke condensate (CSC) induces CYP expression and oxidative stress in HIV-1-infected monocyte-derived macrophages, and the findings are consistent with increased oxidative stress, nicotine metabolism and HIV-1 replication in HIV-infected individuals who smoke^2^. Another study has revealed that the toxic metabolites released through the CYP-mediated metabolism of cigarette smoke constituents enhance HIV-1 gene expression through DNA adduct formation^9^. Aqueous tobacco smoke extract is also known to enhance the upregulation of genes that enhance HIV-1 infection, but downregulate the expression of other genes that promote cell survival and antigen presentation^5^.

Of the 5000 compounds that are present in CSC, polyaryl hydrocarbons (PAHs) are a class of carcinogenic compounds that are implicated by several studies for their potential to induce oxidative stress^10–12^. Benzo(a)pyrene (BaP) is a prototype PAH, which has been widely studied for its carcinogenicity, genotoxicity, and mutagenicity^13–16^. BaP is also known to induce CYP enzymes, especially CYP1A isoforms, which can have a direct impact on the biological disposition of various drugs^17,18^. BaP is metabolically activated by CYP 1A1/1B1 enzymes into epoxide intermediates, which are further metabolized by CYPs or epoxide hydrolase into carcinogenic diol products^19,20^. As a result of CYP-mediated BaP metabolism, excessive reactive oxygen species (ROS) are generated, leading to oxidative stress^21,22^. Oxidative stress further leads to oxidative DNA damage, lipid peroxidation, and the oxidation of several proteins, ultimately causing cytotoxicity and cell death^23,24^. Recently, we have demonstrated that exposure of BaP causes the induction of CYPs and a subsequent increase in oxidative stress and cytotoxicity in U937 monocytic cells^17^.

Oxidative stress is implicated in enhanced replication of HIV-1 via the activation of redox sensitive nuclear transcription factor Kappa- B (NF-κB)^25–28^. Various stress factors regulate the NF-κB pathway resulting in the transcription of over hundreds of genes that regulate inflammation, immune response, cell proliferation, growth, and survival^29–31^. NF-κB is activated by a number of triggers such as viral proteins and drugs of abuse, leading to the expression of various cytokines, chemokines, and CYPs^29,32–34^. Interestingly, most of the stress factors use ROS as a secondary messenger to modulate NF-κB activity^35^. In an inactive state, NF-κB proteins are localized in the cytoplasm by forming a complex with inhibitors of NF-κB proteins (IκB) . ROS triggers the activation of the IκB kinase complex that facilitates the ubiquitination of IκB proteins, thereby releasing the NF-κB proteins into the nucleus^36^. Within the nucleus, the activated NF-κB proteins induce the transcription of HIV-1 structural genes by binding to the enhancer region of long terminal repeat (LTR) on HIV-1 DNA, that contains NF-κB binding sites^37^. Several reports have emphasized the role of ROS in the activation of NF-κB and its subsequent impact on HIV-1 gene transcription^27,38^. However, whether smoking/tobacco mediated oxidative stress via CYP pathways causes the nuclear trafficking of NF-κB and resultant HIV-1 replication, is yet to be examined. In the current study, we examined the potential role of CYP-mediated oxidative stress and subsequent HIV-1 replication via the NF-κB pathway by an important tobacco constituent, BaP, in HIV-1-infected macrophages. We used macrophages in this study because they are a secondary target of HIV-1 infection and a major viral reservoir where it is difficult to effectively suppress the virus with antiretroviral agents^39,40^. Moreover, HIV-1-infected macrophages cross the blood-brain-barrier (BBB) and infect CNS cells such as perivascular macrophages, microglia, and to some extent astrocytes, which eventually cause HIV-1-associated neurocognitive disorders^41,42^.

## Results

### BaP induces HIV-1 replication in U1 cells and HIV-1-infected human primary macrophages

**Figure 1:**
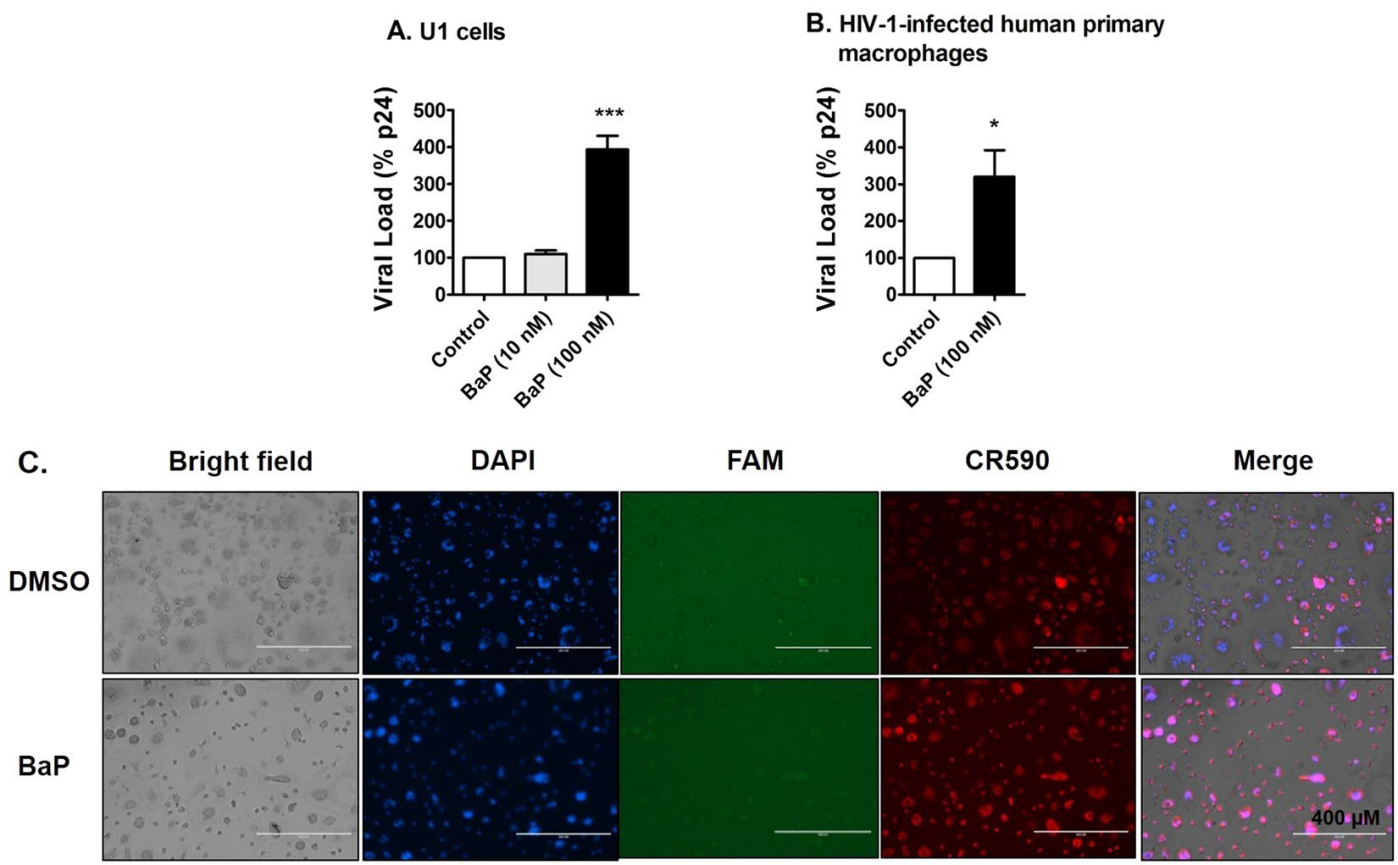
Chronic treatment of BaP induces HIV-1 replication and apoptotic DNA damage in HIV-1-infected macrophages. (**A**) The U1 cells were treated with 10 nM and 100 nM BaP for seven days. After the BaP treatment, the U1 cells were stimulated with 100 nM of Phorbol 12-myristate 13-acetate (PMA) to produce HIV-1. Supernatants were collected after two days of differentiation, which were used for the p24 ELISA assay to assess the viral load. The chronic (7 days) treatment of BaP (100 nM) significantly increased the viral replication in U1 cells, while the 10-fold lower concentration did not have any significant effect. The data is displayed as mean ± SEM (n = 6), calculated as a percentage of the control. (**B**) HIV-infected human primary macrophages were treated with BaP (100 nM) for 3 days. The supernatant was collected thereafter and used for the p24 ELISA assay to assess the viral load. The acute (3 days) treatment of BaP (100 nM) significantly increased the viral replication in HIV-1-infected primary macrophages. The data is displayed as mean ± SEM (n = 4). For calculating the viral load, we subtracted the nonspecific background reading from the actual absorbance values. Since the residual viral load varies from experiment to experiment in U1 cells, we normalized the control values for each experiment to 100% and calculated the values for the treated, as the percentage of the control. The statistical significance was calculated at *p ≤ 0.05, where *** represents p ≤ 0.0005, compared with the control group. (**C**) The apoptotic DNA damage assay was performed on the treated cells. DAPI, FAM and CR590 stained nucleus (blue), apoptotic DNA damage with DNase Type II ends (green) and Type I ends (red) respectively. A higher signal for CR590 is visible in the fluorescent images, indicating apoptotic DNA fragmentation with DNase Type I ends in the infected human primary macrophages after BaP (100 nM) exposure for 3 days. DNA fragmentation with DNase Type II ends (green) was not visible in either the control or the treated cells. Therefore, the images indicate that BaP (100 nM) induces DNA fragmentation during the early phase of apoptosis in the HIV-1-infected human primary macrophages.

Chronic exposure of BaP (100 nM for 7 days) showed an approximately 4-fold increase in HIV-1 replication in U1 cells (Fig. 1A). However, a 10-fold lower concentration of BaP (10 nM) did not have any significant effect on the viral replication. We also confirmed this result in HIV-1-infected human primary macrophages, in which, BaP (100 nM) showed an approximately 3-fold increase in HIV-1 replication (Fig. 1B). Furthermore, we examined apoptotic DNA fragmentation in HIV-1-infected human primary macrophages after 3 days exposure of BaP (100 nM). The fluorescent images revealed an increased apoptotic DNA fragmentation with DNase Type I ends in cells treated with BaP (100 nM) compared to that of the control (Fig. 1C). DNA fragmentation with DNase Type II ends were not visible in both the control and the treated cells. The results suggest that BaP induces DNA fragmentation during the early phase of apoptosis, within the nucleus of the treated cells. Upon validating the results of the U1 cells in human primary macrophages, we performed the subsequent experiments that examined the underlying mechanism in U1 cells.

### BaP induces the expression of CYP1A1

**Figure 2:**
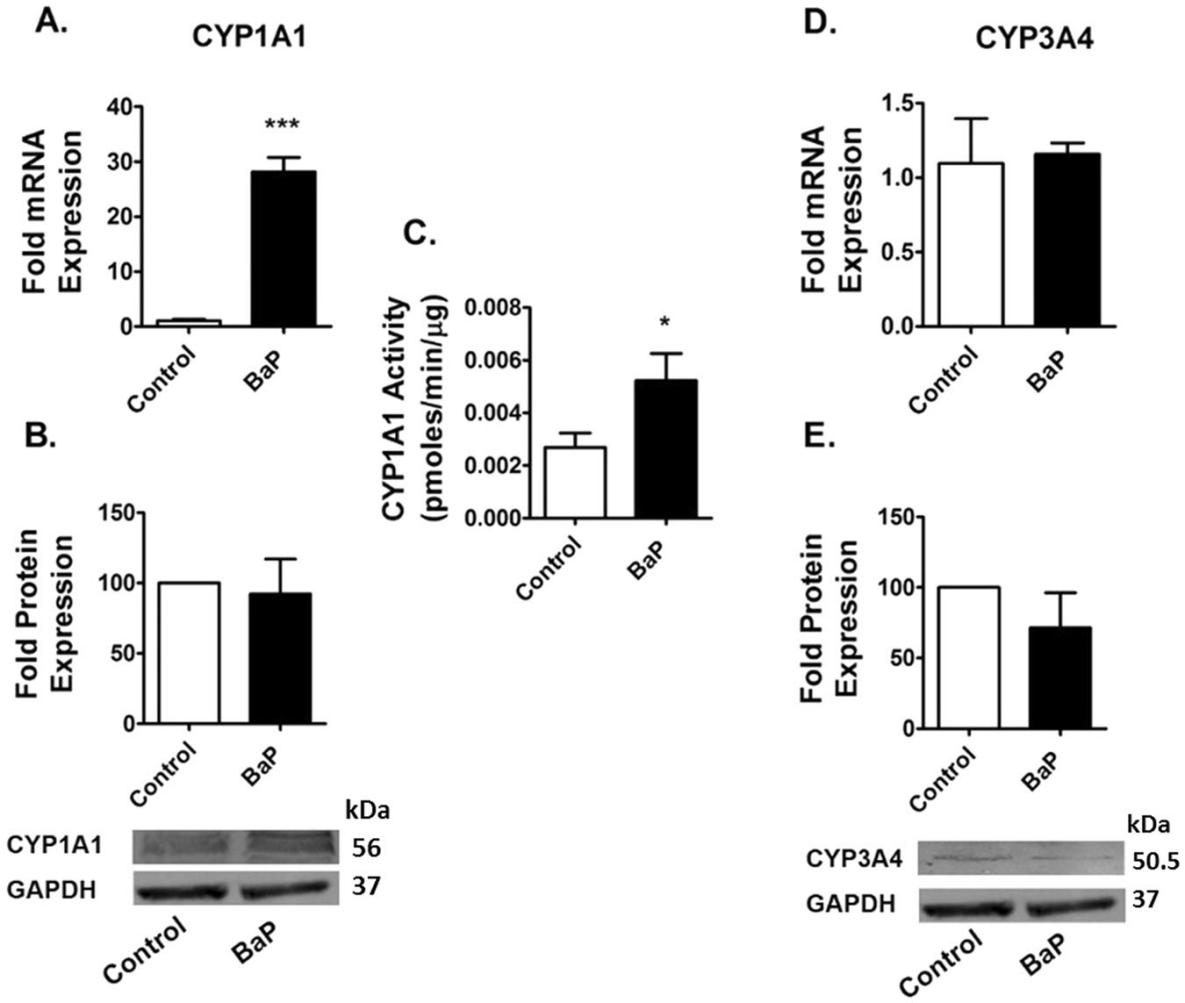
Chronic treatment of BaP induces expression of CYPs in U1 cells. The U1 cells were treated with 100 nM BaP for seven days. We measured the mRNA expression (**A**,**D**) and protein expression (**B**,**E**) of CYPs (1A1, 3A4) using RTPCR and western blotting, respectively. Chronic exposure of BaP (100 nM) significantly induced the expression of CYP1A1 at the mRNA level, but not at the protein level. Therefore, we measured activity of CYP1A1 using the EROD assay (**C**). Chronic BaP treatment increased CYP1A1 activity by approximately 2.5-fold. However, there was no significant change in the expression of CYP3A4 at both the mRNA and protein levels. The data are displayed as mean ± SEM of at least three independent experiments (n ≥ 3). The mRNA/protein expression of the treated cells are normalized to control cells, whose expression was set at 1-fold. GAPDH was used as an endogenous control and loading control for RTPCR and western blotting, respectively. The statistical significance was calculated at *p ≤ 0.05 compared with the control group. The blots are representative of at least three independent experiments.

The expression of CYP1A1 and CYP3A4, which are the major BaP-metabolizing CYPs, were examined in U1 cells exposed to BaP (100 nM) for 7 days. The chronic exposure of BaP showed an approximately 30-fold increase in the mRNA expression of CYP1A1 in U1 cells (Fig. 2A), but failed to show any significant expression of CYP1A1 at the protein level (Fig. 2B). The chronic exposure of BaP (100 nM) increased the enzymatic activity of CYP1A1 by approximately 2.5-fold (Fig. 2C). On the other hand, we did not observe any significant change in the expression of CYP3A4 at both the mRNA and protein levels (Fig. 2D and E).

### BaP does not alter the expression of AOEs

**Figure 3:**
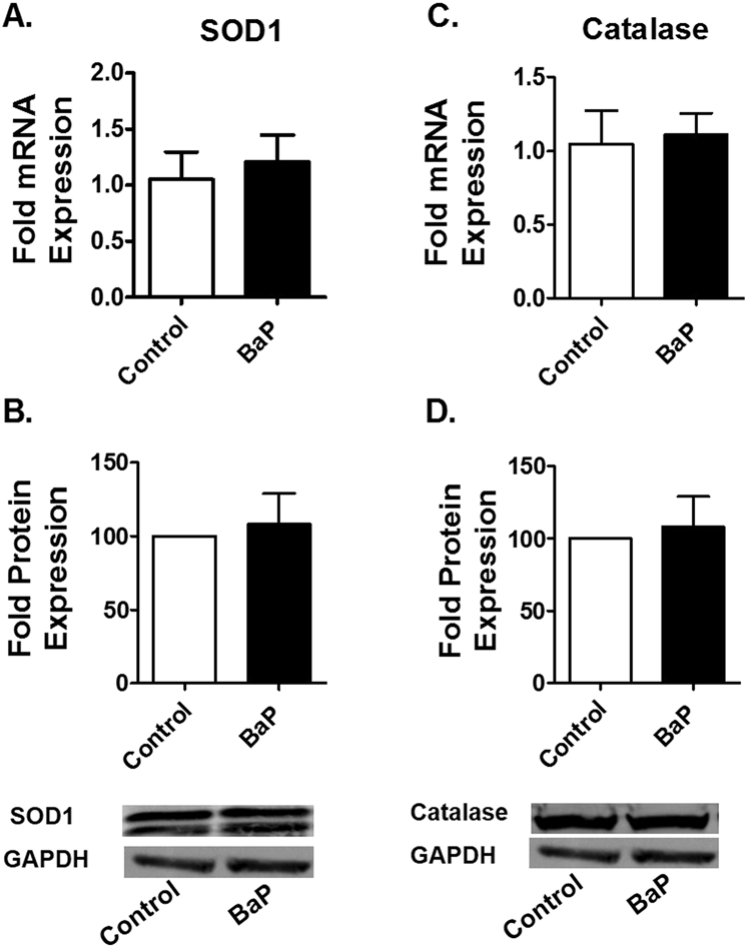
Chronic treatments of BaP have no significant effect on the expression of AOEs in U1 cells. The U1 cells were treated with 100 nM BaP for seven days. We measured the mRNA expression (**A**,**C**) and protein expression (**B**,**D**) of AOEs (SOD1 and Catalase) using RTPCR and western blotting, respectively. There was no significant change in the expression of SOD1 and catalase at both the mRNA and protein levels after chronic treatment of BaP (100 nM). The data are displayed as mean ± SEM of at least three independent experiments (n ≥ 3). The mRNA/protein expression of treated cells are normalized to control cells, whose expression was set at 1-fold. GAPDH was used as an endogenous control and loading control for RTPCR and western blotting, respectively. The statistical significance was calculated at *p ≤ 0.05 compared with the control group. The blots are representative of at least three independent experiments.

The induction of CYPs could metabolize BaP and increase ROS. However, the ROS could subsequently be neutralized by the induction of antioxidant enzymes (AOEs). Therefore, we examined the induction of two of the most important and general AOEs; superoxide dismutase 1 (SOD1) and catalase by 100 nM BaP. Our results demonstrated no significant change in the mRNA and protein expression levels of both SOD1 and catalase (Fig. 3A–D).

### Role of CYP1A1 in BaP-induced ROS generation

**Figure 4:**
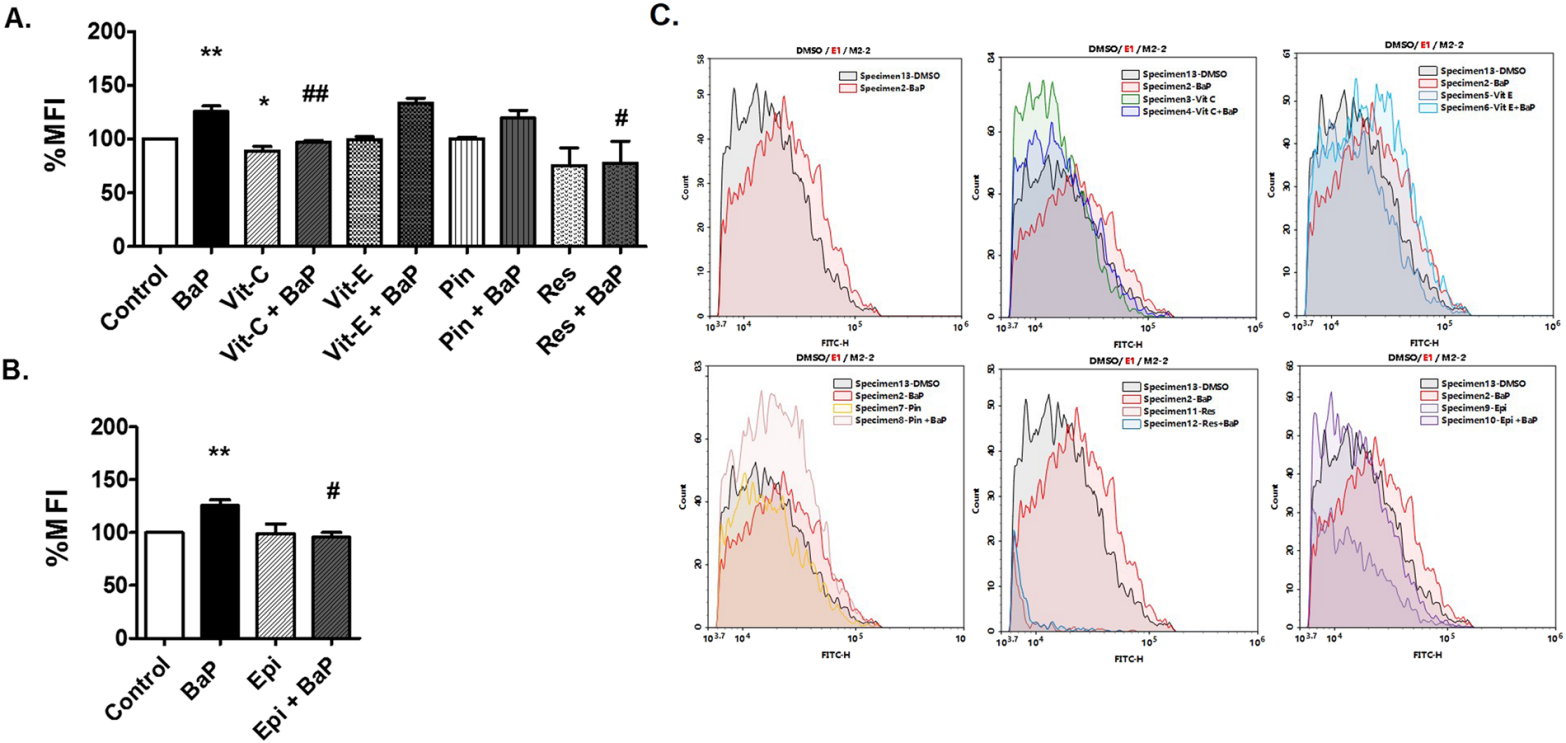
Treatment of antioxidants and CYP1A1 inhibitors reduce ROS in U1 cells due to BaP exposure. U1 cells were concomitantly treated with BaP (1 µM) and antioxidants [vitamin C (Vit-C, 100 µM), vitamin E (Vit-E, 100 µM), pinostilbene (Pin, 2 µM), and resveratrol (Res, 50 µM)] (**A**) or a CYP1A1 inhibitor [ellipticine (Epi, 1 µM)]. (**B**) Treated cells were stained with CM-DCFDA dye and the fluorescence emitted was measured using flow cytometry at excitation/emission of 495/519 nm. Treatment of BaP (1 µM) significantly increased ROS in U1 cells, which was rescued by the treatment of vitamin C, resveratrol and ellipticine. The data were obtained from the mean of at least three independent experiments. * and ** represents p ≤ 0.05 and p ≤ 0.005 respectively, compared with the control group while ^#^ and ^##^ represents p ≤ 0.05 and p ≤ 0.005, respectively, compared to the BaP-treated groups. Figure **C**. shows the graphical representation of mean fluorescence intensity due to the treatments.

Treatment of BaP (1 µM) in U1 cells for 3 days increased the generation of ROS by 30%. In this and the subsequent experiments we used 1 µM BaP for 3 days to induce oxidative stress and/or HIV-1 replication, because chemical inhibitors and antioxidants are toxic to the cells when treated for 7 days. Further, we pre-treated the U1 cells with different antioxidants or CYP inhibitors prior to BaP exposure to determine whether these compounds reduce the BaP-induced ROS generation. We used vitamin C (100 µM), vitamin E (100 µM), resveratrol (50 µM), a resveratrol analog, pinostilbene (2 µM) as antioxidants, and a selective CYP1A1 inhibitor ellipticine (1 µM) for the study at the concentrations previously shown to be effective^43,44^. Vitamin C, resveratrol, and ellipticine significantly reduced the BaP-induced production of ROS (Fig. 4). However, there was no significant change in BaP-induced ROS after treatment with vitamin E, and pinostilbene.

### Role of CYP1A1 and oxidative stress pathways in BaP-induced HIV-1 replication

**Figure 5:**
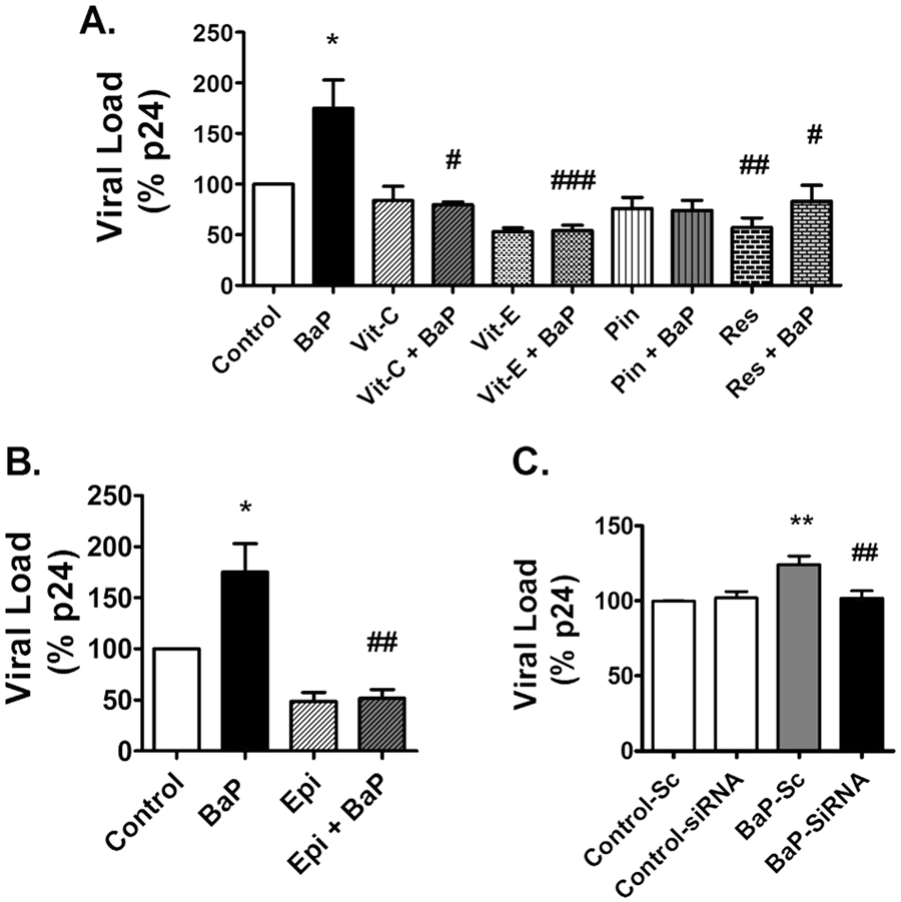
Treatment of antioxidants and CYP1A1 inhibitors reduce HIV-1 replication in U1 cells due to BaP exposure. U1 cells were concomitantly treated with BaP (1 µM) and antioxidants [vitamin C (100 µM) and vitamin E (100 µM), pinostilbene (2 µM), and resveratrol (50 µM)] (**A**) or CYP1A1 inhibitor ellipticine (1 µM)] (**B**) for 3 days. Prior to BaP treatment, the CYP1A1 gene was knocked down in the U1 cells using siRNA specific to CYP1A1. (**C**) The cells were then treated with BaP (100 nM) for 3 days. After the treatment, supernatants were collected to determine the viral load using the p24 ELISA assay. HIV-1 replication significantly increased with 3-days exposure of BaP (1 µM), which was rescued by all the antioxidants (vitamin C and E, and resveratrol) as well as the CYP1A1 inhibitor, ellipticine . The knock-down of the CYP1A1 gene also rescued HIV-1 replication in BaP-exposed U1 cells. The data were obtained from the mean of at least three independent experiments. * and ** represents p ≤ 0.05 and p ≤ 0.005 compared with the control group while ^#,##^ and ^###^ represents p ≤ 0.05, p ≤ 0.005 and p ≤ 0.0005, respectively, compared to the BaP-treated groups.

The U1 cells treated with BaP (1 µM) for 3 days showed approximately 70% increase in HIV-1 replication (Fig. 5A). Treatment with antioxidants, vitamin C and E (100 µM each) and, resveratrol (50 µM) rescued the viral replication caused by BaP (1 µM) (Fig. 5A). In addition, treatment with the CYP1A1 inhibitor, ellipticine (1 µM) also exhibited a reduction in viral load in BaP-treated U1 cells (Fig. 5B). To further validate our results, we knocked down the CYP1A1 gene in the U1 cells, using a siRNA silencing technique prior to BaP treatment. The viral load significantly decreased after silencing the CYP1A1 gene (Fig. 5C), which further confirms a role of CYP1A1 on BaP-induced HIV-1 replication.

### Nuclear translocation of NF-κB subunits upon BaP exposure

**Figure 6:**
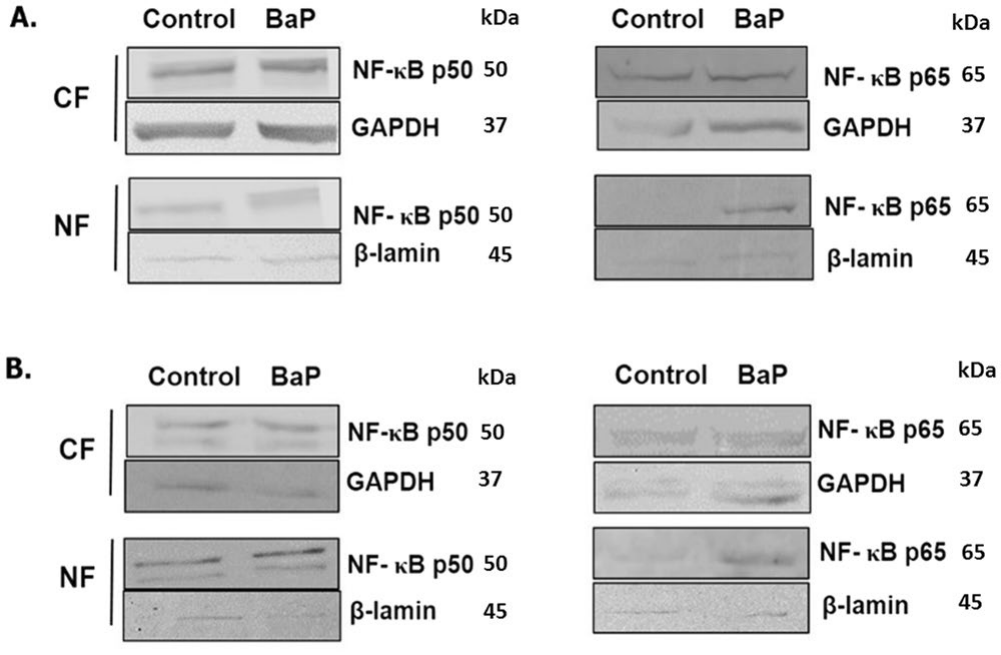
Translocation of NF-κB subunits from cytoplasm to nucleus upon BaP exposure. U1 cells were treated with BaP 100 nm (**A**) and 1 µM (**B**) for 7 days and 3 days, respectively. After the treatment, proteins from the cytoplasm and nucleus were extracted from the cells. Western blot was run to determine the expression of the NF-κB p50 and p65 subunits in the proteins in cytosolic fraction (CF) and nuclear fraction (NF). GAPDH and β-lamin were used as loading controls for the cytoplasmic and nuclear proteins, respectively. The blots are representative of at least three independent experiments. There is not much difference in the expression of NF-κB p50 and p65 between the control and the BaP-treated cells in the cytoplasmic fraction. However, there is a clear increase in the expression of both the subunits in the nuclear fraction of acutely or chronically BaP-treated cells compared to the control group.

There was no prominent change in the expression level of NF-κB p50 and p65 in the cytoplasmic fraction after chronic (Fig. 6A) and acute (Fig. 6B) treatment of BaP (100 nM) and BaP (1 µM), respectively. Interestingly, there was an increase in the expression of both the NF-κB proteins, especially p65, in the nuclear fraction after acute and chronic BaP exposures compared to the control (Fig. 6A and B).

### Role of NF-κB pathway in BaP-induced HIV-1 replication

**Figure 7:**
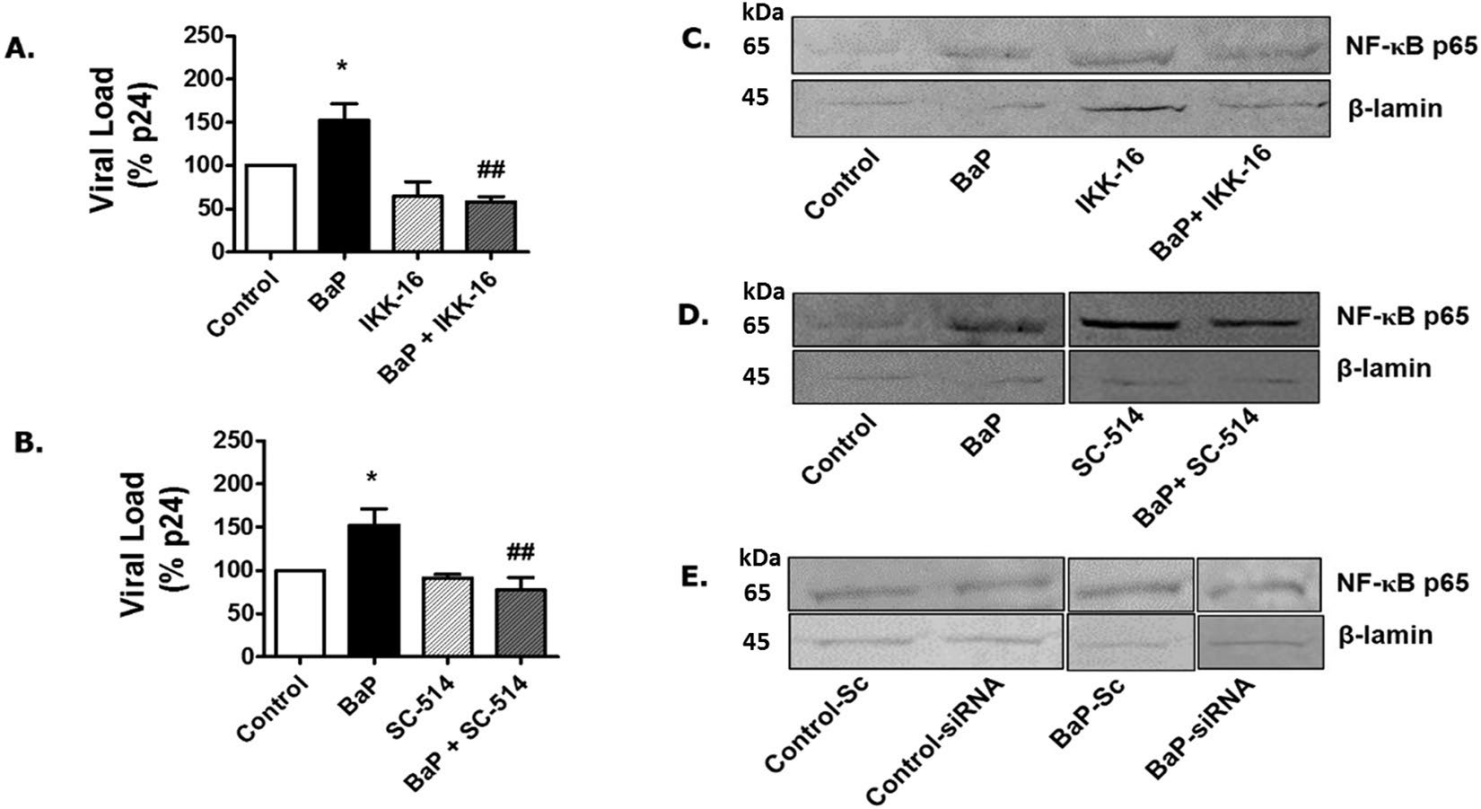
Treatment of NFκ-B inhibitors reduce HIV-1 replication in U1 cells due to BaP exposure. U1 cells were concomitantly treated with BaP (1 µM) and NFκ-B inhibitors, IKK-16 (400 nM) (**A**), and SC-514 (10 µM) (**B**) for 3 days. After the treatment, supernatants were collected to determine the viral load using the p24 ELISA assay. HIV-1 replication due to BaP (1 µM) exposure was significantly rescued by NFκ-B inhibitors, IKK-16 (400 nM) and SC-514 (10 µM). *Represents p ≤ 0.05 compared with the control group while ^##^ represents p ≤ 0.005 compared to the BaP-treated groups. Western blots were run using the nuclear fraction proteins obtained from the BaP-exposed cells treated with IKK-16 (**C**) SC-524 (**D**) or siRNA CYP1A1 (**E**) to determine the expression of NFκ-B p65 subunits. The blots indicate that treatment with both the NFκ-B inhibitors and CYP1A1 siRNA reduced the expression of NFκ-B p65 in the nuclear fraction protein of the BaP-treated cells compared to the control. The blots presented are representative of at least three different experiments.

Treatment of BaP (1 µM)-exposed U1 cells with NF-κB inhibitors such as IKK-16 (Fig. 7A) or SC-514 (Fig. 7B) for 3 days, significantly reduced HIV-1 replication in BaP-exposed U1 cells, suggesting that the viral replication occurred via the NF-κB signaling pathway. We also monitored the translocation of NF-κB p65, the major DNA-binding subunit of the NF-κB protein into the nucleus, after treatment with NF-κB inhibitors, IKK-16 (Fig. 7C) and SC-514 (Fig. 7D) and after siRNA silencing of CYP1A1 (Fig. 7E). The results showed that the knock-down of CYP1A1 in BaP-treated cells reduces the translocation of the NF-κB p65 unit into the nucleus. As expected, the expression of the NF-κB p65 protein was also observed to be lower in the nucleus of BaP-treated cells, after treatment with IKK-16 and SC-514.

## Discussion

**Figure 8:**
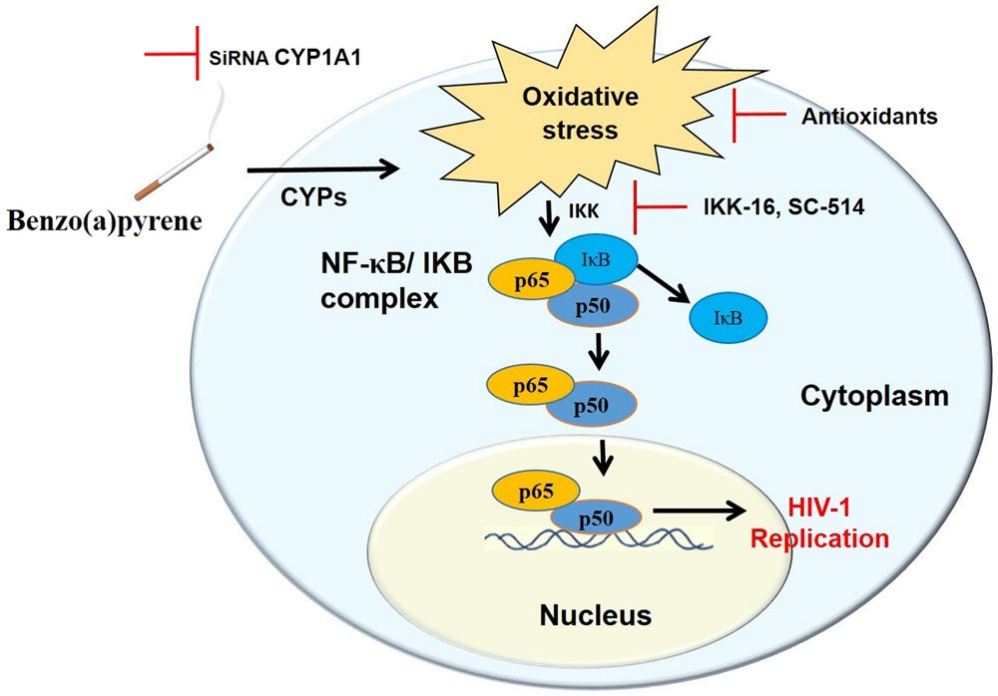
Schematic diagram for the mechanism showing BaP-mediated HIV-1 replication in U1 cells. Cigarette smoke constituents such as benzo(a)pyrene (BaP) induces expression of CYP1A1 in HIV-1-infected cells. CYP1A1 in turn metabolizes BaP into various BaP-metabolites, during which large amounts of reactive oxygen species (ROS) are generated. The resulting oxidative stress induces HIV-1 replication in the cells via a NF-κB pathway. In an inactive state, NF-κB (p65 and p50 subunits) forms a complex with an inhibitor of NF-κB proteins (IκB), which inhibits its translocation from the cytoplasm to the nucleus. Oxidative stress due to BaP triggers the activation of IκB kinase (IKK) that facilitates the ubiquitination of IκB proteins, thereby releasing the NF-κB subunits into the nucleus. Within the nucleus, the NF-κB proteins bind to specific DNA regions and trigger the expression of HIV-1 structural genes. Knock-down of CYP1A1 by siRNA specific to CYP1A1 and inhibition of the NF-κB pathway using specific NF-κB inhibitors such as IKK-16 and SC-514 significantly reduced HIV-1 replication in HIV-1-infected macrophages.

Several reports suggest that cigarette smoke exposure is associated with increased HIV-1 replication and infectivity^5,45,46^. However, the mechanism of smoking induced HIV-1 replication is poorly understood, except for the fact that oxidative stress is a possible mechanism for enhanced viral load^2,3^. The current report reveals a novel mechanism for BaP-mediated HIV-1 replication in monocyte-derived macrophages *in vitro*. In this study, we have demonstrated that oxidative stress generated by the CYP1A1-mediated metabolism of BaP, triggers the redox-sensitive transcription factor, NF-κB that leads to the amplification of HIV-1. This is the first evidence of the involvement of a novel CYP-mediated oxidative stress pathway in tobacco-mediated HIV-1 replication via NF-κB in macrophages (Fig. 8).

Recently, we have shown that the exposure of CSC increases HIV-1 replication, oxidative stress, and induction of CYP enzymes in U937 and/or U1 macrophages^2^. We have also shown the involvement of CYP2A6 in nicotine metabolism and oxidative stress in U937 cells^47^. Further, our study using *ex vivo* samples from HIV-1-infected individuals who smoke also demonstrated an increase in HIV-1 replication, oxidation stress, and nicotine metabolism^3^. Taken together, these studies suggest an association of CYP-mediated oxidative stress with HIV-1 replication in tobacco smokers. Comparison of oxidative stress levels after nicotine and CSC exposure revealed that the induction of ROS by CSC is much higher than ROS induction by nicotine^47^. Therefore, we recently studied the effect of BaP, an important PAH component of CSC, in U937 cells and observed that BaP increases CYP1A1 expression, ROS levels, and cytotoxicity^17^. The excessive ROS production by BaP likely disturbed the redox homeostasis, causing oxidative stress, which resulted in cytotoxicity in U937 cells^48^.

In the present study, we examined whether BaP induces HIV-1 replication in HIV-1-infected macrophages via the CYP-induced oxidative stress pathway. To demonstrate this, first we studied the effect of BaP in HIV-1 replication in U1 cells and our findings suggested that chronic exposure of BaP (100 nM) increases HIV replication in these cells. We have also confirmed this result in HIV-1-infected human primary macrophages. However, treatment of BaP (100 nM) acute exposure (3 days) in primary macrophages shows relatively lower viral expression (~3-fold) (Fig. 1B) compared with chronic exposure (7 days) in U1 cells (~4-fold) (Fig. 1A). Furthermore, acute (3 days) exposure of BaP at relatively high concentration (1 µM) in U1 cells resulted in ~ 1.5- to 1.75-fold increase in p24 levels (Figs 5A,B and 7A,B). This variation in p24 levels is possibly due to exposure of different concentration of BaP (100 nM or 1 µM) for a different exposure time (3 days or 7 days) in different cell types (U1 cells or HIV-1-infected primary macrophages). For mechanistic study, we optimized experiments with U1 cells for shorter exposure time at relatively higher BaP concentration to minimize the effect of other agents alone such as antioxidants and CYP inhibitors.

Furthermore, BaP exposure induced DNA fragmentation during the early phase of apoptosis. Secondly, the expression of CYP1A1 and CYP3A4, which are the major BaP-metabolizing CYPs, were examined in U1 cells after BaP (100 nM) exposure for 7 days. The results showed an increase in the mRNA expression of CYP1A1 by BaP which is consistent with the previous study findings^17,19,49,50^. BaP induces the expression of CYPs in cells by activating a nuclear receptor, aryl hydrocarbon receptor (AHR)^51^. The binding of BaP dissociates AHR from the AHR-heat shock protein 90 complex, which facilitates AHR translocation into the nucleus. Inside the nucleus, AHR binds to DNA with the help of AHR nuclear translocator (ARNT) and activates the transcription of CYP1A1, CYP1A2 and CYP1B1^52,53^. This is a well-known pathway and therefore a similar mechanism likely occurs in inducing CYP1A1 by BaP in macrophages presented in this study. However, as seen before, we did not observe any significant expression of CYP1A1 at the protein level, perhaps due to post translational modifications of the protein or instability of the protein after extraction. Therefore, we determined its enzymatic activity using the Erod assay^54^. The chronic exposure of BaP (100 nM) increased the enzymatic activity of CYP1A1 by approximately 2.5-fold. The substrate and the cofactors added during the enzymatic reaction perhaps enhanced the stability of the CYP1A1, thereby increasing its enzymatic activity. On the other hand, we did not observe any significant change in the expression of CYP3A4 at both the mRNA and protein levels. However, BaP induced the expression of CYP3A4 in U937 cells^17^. This discrepancy could be due to the use of two different, uninfected (U937) and HIV-infected (U1), monocytic cell lines.

Thirdly, it is well known that CYPs generate ROS while metabolizing a wide range of substrates via coupling and auto oxidation reactions^55^, and our results are in agreement with previous reports^22,56^ showing that acute treatment of BaP (1 µM) in U1 cells for 3 days increase the ROS levels. Further, we treated the U1 cells with different antioxidants or CYP inhibitors prior to BaP exposure to determine whether these compounds reduced the BaP-induced ROS generation. We chose a shorter duration of time and a higher BaP concentration to induce ROS in this experiment, because chronic treatment of antioxidants or CYP inhibitors along with BaP causes cytotoxicity. We used vitamin C, vitamin E, resveratrol, a resveratrol analog, pinostilbene as antioxidants, and a selective CYP1A1 inhibitor ellipticine to see if these antioxidants can block the BaP-induced oxidative stress. In addition to their antioxidant effects, resveratrol and pinostilbene are also known to inhibit CYP1A1^44^. Vitamin C, resveratrol and ellipticine significantly reduced the BaP-induced production of ROS. The results suggest that BaP-induced ROS occurs via CYP1A1-mediated metabolic activation of BaP.

Cells express AOEs to neutralize the excessive accumulation of ROS generated by various stress factors^24,57^. BaP is known to induce the expression of AOEs by the activation of the Nrf2 (nuclear factor erythroid 2-related factor) pathway^58^. BaP downregulates the activity of the Nrf2 inhibitory protein, kelch-like ECH-associated protein 1(*Keap1*), which prevents the proteasomal degradation of Nrf2 and promotes its translocation to the nucleus, where it binds to the enhancer ARE in DNA, leading to the transcription of the AOEs^58,59^. Therefore, we examined the induction of two of the most important and general AOEs; superoxide dismutase 1 (SOD1) and catalase in U1 cells after BaP exposure. There was no significant change in the mRNA and protein expression levels of both SOD1 and catalase. Although the basal AOEs may have played their part in scavenging ROS, the persistent increase in oxidative stress after BaP exposure suggests that their antioxidant capacity was not sufficient. Furthermore, these results suggest that the inability of BaP to enhance AOE expression could cause an increased generation of CYP-induced ROS by BaP.

Next, we checked whether oxidative stress induced through the CYP1A1-mediated metabolism of BaP increased HIV-1 replication in HIV-1-infected macrophages. The treatment of U1 cells with BaP showed an approximately 70% increase in HIV-1 replication, which was significantly reduced by the treatment of antioxidants such as vitamin C and E, and resveratrol. In addition, treatment with the CYP1A1 inhibitor, ellipticine, also exhibited a reduction in the viral load of BaP-treated U1 cells. Taken together, these results suggest that BaP induces HIV-1 replication in U1 cells via the CYP-mediated oxidative stress pathway. We knocked down the CYP1A1 gene in the U1 cells, using the siRNA silencing technique prior to BaP treatment to further confirm our results. The reduction in HIV-1 replication after siRNA silencing of the CYP1A1 gene, further strengthens the role of CYP1A1 in BaP-induced HIV-1 replication.

Upon demonstrating that BaP enhances HIV-1 replication in U1 cells via the CYP-mediated oxidative stress pathway, our next goal was to identify the molecular mechanism of oxidative stress-induced viral replication upon BaP exposure. Activation of NF-κB pathway by cigarette smoke has been observed in various cell types such as monocytic cells (U937), T cells (Jurkat), lung cells (H1299), and head and neck squamous cell lines (1483 and 14B)^60^. Cigarette smoke is known to activate NF-κB by inducing the phosphorylation of IκB, an inhibitor of NF-κB, which causes degradation of IκB allowing the NF-κB subunits to translocate from cytoplasm to nucleus^60–62^. Furthermore, several studies have suggested that the NF-κB pathway triggered by ROS has a role in HIV-1 replication^26,28^. Having shown that CYP1A1-mediated metabolism of BaP generates ROS, we were then interested to see if the oxidative stress thus generated was responsible for NF-κB activation and the subsequent HIV-1 replication.

Therefore, we monitored the translocation of NF-κB subunits from the cytoplasm into the nucleus after chronic treatment of BaP in U1 cells. Since the differentiated macrophages specifically express the transcriptionally active NF-κB p65-p50 heterodimer^63^, and this heterodimer is specifically expressed during NF- κB-mediated HIV-1 transcription^64^, we monitored the protein expression of these NF-κB subunits in both the cytoplasm and the nucleus. We observed that BaP increases the expression of both of the NF-κB proteins, especially p65, in the nuclear fraction after acute and chronic BaP exposure. Based on these findings, we suggest that ROS generated through CYP1A1-mediated metabolism of BaP could trigger the NF-κB pathway, which eventually perpetuates viral transcription. Furthermore, we confirmed the involvement of the NF-κB pathway in BaP-induced HIV-1 replication by treating BaP-exposed U1 cells with NF-κB inhibitors such as IKK-16 and SC-514. Both of the compounds inhibit NF-κB activity by selectively acting on IKK-β, an isoform of IKK protein^65,66^. In addition, SC-514 decreases the import of p65 into the nucleus and expedites the export of p65 from the nucleus, as well as inhibits the phosphorylation and transactivation of p65^67^. Treatment of IKK-16 or SC-514 significantly reduced HIV-1 replication in BaP-exposed U1 cells, which provides strong evidence that the viral replication occurred via the NF-κB signaling pathway. To further verify our results, we monitored the translocation of NF-κB p65, the major DNA-binding subunit of the NF-κB protein into the nucleus, after treating the cells with the NF-κB inhibitors, IKK-16 and SC-514 and after siRNA silencing of CYP1A1. The knock-down of CYP1A1 in BaP-treated cells reduced the translocation of the NF-κB p65 unit into the nucleus, which shows an association of CYP1A1 with NF-κB-mediated viral replication. As expected, the expression of the NF-κB p65 protein was also observed to be lower in the nucleus of BaP-treated cells, after treatment with IKK-16 and SC-514, which further confirms that BaP-mediated HIV-1 replication occurs via the NF-κB signaling pathway. Our findings reconfirm the role of ROS in the activation of NF-κB and its subsequent impact on HIV-1 gene transcription^68,69^.

In addition to the ROS generated via the CYP-mediated metabolism of BaP, there are other factors such as HIV-1 proteins whose contribution to ROS production cannot be ruled out. HIV-1 proteins such as tat, gp120, Nef, and Vpr are known to cause oxidative stress in the infected cells^70–74^. These HIV-1 proteins generate ROS via different mechanisms: tat via upregulation of spermine oxidases^75^ or by activating the NADPH oxidase pathways^76^; gp120 via upregulation of CYP2E1, NADPH oxidases^72^, and proline oxidase^77^; Vpr via interaction with adenine nucleotide translocator or NADPH oxidases^73^; and Nef via direct interaction with NADPH oxidases^78^. Moreover, some of these viral proteins, such as tat and nef, are also known to enhance HIV-1 replication by interacting directly or indirectly on the LTR of the viral DNA^79^.

In conclusion, the present study suggests NF-κB activation through ROS generated via CYP1A1-mediated activation/metabolism of BaP as a novel pathway to explain smoking-mediated HIV-1 replication in monocytes-derived macrophages. This study has clinical relevance because the outcomes obtained from this study provide potential targets such as CYPs, oxidative stress, and NF-κB signaling pathways, for developing novel interventions to improve treatment strategies for HIV-1-infected smokers. We have demonstrated that antioxidants such as vitamin C and E, resveratrol, and CYP1A1 inhibitors namely ellipticine are capable of neutralizing the oxidative stress induced by BaP and subsequent viral load. These antioxidants and CYP1A1 inhibitors, which are chemodietary agents, have the potential to effectively control viral replication in HIV-1-infected individuals who smoke tobacco. Furthermore, reduction of the viral load in these cells by targeting CYPs and the oxidative stress pathway may be beneficial in treating HIV-infected CNS cells.

## Materials and Methods

### Cell culture and treatment

#### U1 cells

U1 cells, which are U937 cells chronically infected with HIV-1, were obtained from the NIH AIDS Reagent Program (Germantown, MD). The cells were cultured in Roswell Park Memorial Institute (RPMI) 1640 media containing 10% Fetal bovine serum (FBS) and penicillin. To differentiate the cells into macrophages, 0.8 million cells were seeded in 1.5 ml of media containing 100 nM phorbol 12-myristate 13-acetate (PMA) in each well of a 6-well plate. After 3 days, the media containing PMA and non-adherent cells was removed and the differentiated cells were washed with phosphate buffer saline (PBS). The cells were topped with fresh 1 ml media and treated with BaP (1 µM) every 24 hours for 3 days for acute treatment. An additional 0.5 ml of media was added and BaP concentration was maintained constant at every treatment. In order to monitor the chronic (7 days) effect of BaP, we initially treated U1 cells with BaP (10–100 nM) for 7 days and later differentiated them into macrophages.

#### Human primary macrophages infected with HIV-1

Peripheral blood mononuclear cells (PBMC) were isolated from buffy coats (60 ml) obtained from the Interstate Blood Bank Inc., Memphis, TN, using density gradient fractionation as described previously^2^. Briefly, in order to isolate the monocytes from the whole blood, we added RosetteSep Human Monocyte Enrichment Cocktail (Stem Cell Technologies, Seattle, WA) to the whole blood and layered it on the top of Ficoll and centrifuged at 1200 g for 20 minutes. The monocyte enrichment Cocktail contains tertrameric antibody complexes that bind non-monocytic cells and red blood cells, and pellet them when centrifuged over a buoyant centrifuge medium. The purified layer of monocytes appeared as a white ring between the plasma and the ficoll layers. The layer of monocytes was carefully isolated and washed a few times with PBS and incubated with Ammonium-Chloride-Potassium (ACK) lysing buffer (Thermo Fischer Scientific, Rockford, IL) to lyse red blood cells. The purified monocytes were cultured in RPMI media supplemented with human serum and macrophage colony stimulating factor (MCSF) (50 ng/ml) to promote differentiation into macrophages. After the monocytes differentiated into macrophages in 7–10 days, they were treated with polybrene (2 µg/ml) and IL-2 (interleukin-2, 10 ng/µl) and infected with HIV-Ada strain (20 ng/10^6^). The infected cells were then treated with BaP (100 nM) every 24 hours for 7 days.

#### Viral Load

The viral load of HIV-1 was determined by assessing the level of p24 antigen in the supernatant collected from the treated U1 cells and primary macrophages. We used the HIV-1 p24 Antigen ELISA kit (Zeptometrix Corporation, Buffalo, NY) for this purpose. The kit is comprised of monoclonal antibody- coated microwells, which specifically bind the HIV-1 p24 antigen in the added samples. The captured antigen was incubated with biotin conjugated human anti-HIV-1 antibody at 37 °C for 1 hr, followed by incubation with enzyme, streptavidin-peroxidase and tetramethylbenzidine substrate at 37 °C and room temperature/dark, respectively, for 30 minutes each. The reaction of the enzyme with the substrate developed a blue color, the absorbance of which was measured at 450 nm to determine the p24 level. The optical density of the samples was compared against the standard curve.

### Isolation of RNA and protein

RNA was isolated using RNeasy Mini kit (250) (QIAGEN, Germantown, MD), following the manufacturer’s protocol. The extracted RNA was quantified using Nanodrop 2000c Spectrophotometer (Thermo Fisher Scientific) at 260 nm. To isolate the protein from the treated cells, 100 µl of RIPA buffer was added to the cell pellet. The cell suspension was sonicated for 30 seconds with pulse set at 4 and centrifuged at 13000 rpm for 5 minutes. The supernatant containing the protein was collected and protein quantification was done by using the BCA protein assay kit (Thermo Fischer Scientific).

### Quantitative reverse transcriptase polymerase chain reaction (RTPCR)

The relative mRNA fold expression level of CYPs and AOEs after chronic BaP treatment was monitored using quantitative RTPCR as described in our previous studies^17^. Initially, 120 ng purified RNA from the cells were reverse transcribed to cDNA using SimpliAmp Thermal Cycler (Applied Biosystems, Foster City, CA). The cDNA was amplified using Taqman Gene Expression kit (Applied Biosystems) in a Step-One Plus Real-Time PCR System (Applied Biosystems). We determined the mRNA expression level of CYPs and AOEs using the following probes from Applied Bioscience: CYP1A1 (Hs01054794_m1), CYP3A4 (Hs00430021_ml), SOD1 (Hs00533490_ml) and catalase (Hs00156308_ml). To calculate mRNA fold expression of the genes, we used the 2^−ΔΔCt^ method and glyceraldehyde 3-phosphate dehydrogenase (GAPDH) as an endogenous control.

### Western Blotting

We determined the protein expression in the treated cells using Western blotting. Approximately 30 µg of protein in 5% SDS was loaded into a polyacrylamide gel (4% stacking, 10% resolving gel). The gel was run for 90 minutes at 150 V, which separated the proteins based on their molecular weight. The proteins from the gel were transferred to a polyvinyl fluoride membrane using a current of 0.35 Amp for 90 minutes. After the proteins were transferred to the membrane, it was blocked with 5–10 ml of Li-Cor blocking buffer (LI-COR Biosciences, Lincoln, NE) for 1 hour to prevent the nonspecific binding of antibodies to its surface. The membrane was then incubated with primary antibodies (GAPDH Rabbit Mab, 1:2000 dilution, Cell Signaling Technology, Danvers, MA; CYP1A1 rabbit Mab, 1:200 dilution, Abcam, Cambridge, MA; CYP3A4 Mouse Mab. 1:200 dilution; SOD1 Mouse Mab, 1:1500 dilution; Catalase Mouse Mab, 1:1200 dilution; NFκB p50 Rabbit Mab, 1:200 dilution, NFκB p65 Mouse Mab, 1:200, β-lamin Rabbit Mab, 1:400 dilution, Santa Cruz Biotechnology. Inc. Dallas,TX) at 4 °C. The blots were washed with PBS containing 0.2% Tween-20 several times before incubating with the corresponding secondary antibodies (Goat anti-Mouse Mab, Goat anti-Rabbit Mab, 1:10000 dilution, LI-COR Biosciences) for 1 hour at room temperature away from light. The blots were scanned using Image Studio Lite version 4.0 in a Li-Cor Scanner (LI-COR Biosciences). The fold change in the expression of proteins was calculated based on densitometry data obtained from the Image Studio Lite software. GAPDH was used as an internal loading control to normalize the amount of the proteins used.

### Measurement of ROS level

We quantified ROS generated after acute treatment of BaP on U1 cells using the NovoCyte flow cytometer (ACEA, Biosciences Inc., San Diego, CA). After the acute treatment, the cells were washed with PBS and incubated with 2–5 μL of the fluorescence dye 5-(and-6)-chloromethyl 2′,7′-dichlorodihydrofluorescein diacetate (CM-H_2_DCFDA) (Life Technologies, Oregon, USA) in 1 ml of PBS at 37 °C away from light. CM-H_2_DCFDA diffuses into the live cells, where its acetate and chloromethyl groups react with intracellular esterases and thiol groups. The resulting products generates a fluorescence adduct called dichlorodihydrofluorescein (DCF) when oxidized by the ROS present within the cells. The level of ROS within the cells was quantified by measuring the fluorescence excitation/emission of DCF at 495/519 nm. The data were analyzed by using the NovoExpress software.

### 7-Ethoxy-resorufin-O-deethylase (EROD) assay

We used the EROD assay to determine the activity of CYP1A1 after the chronic treatment of BaP as described previously^54^. Briefly, the treated cells were washed with PBS and suspended in 900 µl 0.1 M Hepes buffer, pH 7.4. The cell suspension was then sonicated for 10 seconds, 3 times, with pulse on and off. The cell extract (90 µL) thus obtained was added with 1 µl of substrate, 7-ethoxyresorufin (100 µM) and incubated at 37 °C for 10 minutes. Later, 10 µl NADPH (10 mM) was added to the reaction mixture and incubated for another 15 minutes. The reaction was stopped by using 75 µl fluorescamine solution in acetonitrile (150 µg/ml). When the cell extracts were incubated with the substrate and NADPH, the CYP1A1 enzyme present in the cells reacted with the substrate resulting in the formation of a fluorescence product, resorufin. The fluorescence thus generated was measured at excitation/emission wavelengths of 535/590. The CYP1A1 activity was calculated by measuring the amount of fluorescence generated per unit time. The quantity of resorufin in the samples was calculated by comparing it with the standard calibration curve prepared by using 0–100 pmol of resorufin standard.

### Apoptotic DNA damage

We used the Apoptag® Iso Dual Florescence Apoptosis Detection kit (Millipore Sigma, Burlington, Massachusetts) to determine apoptotic DNA damage after BaP treatment in U1 cells. The assay was carried out using the manufacturer’s protocol.

### Statistical analysis

All the data are presented as Mean ± SEM of at least three independent experiments. The mean value obtained for the control group was normalized to 100% or 1-fold, to which the treated cells were compared as a % or fold of control, respectively. Student’s t-test or one- or two-way ANOVA were used to calculate the statistical differences (p ≤ 0.05) between the control and the treated groups, where applicable. All the statistical analyses were performed using GraphPad Prism 7 (San Diego, CA).
